# SINEUP-Mediated Overexpression of Endogenous α-Amylase as a Therapeutic Approach in Lafora Disease

**DOI:** 10.3390/genes17030321

**Published:** 2026-03-16

**Authors:** Lorenzo Allegri, Federica Baldan, Catia Mio, Valentina Imperatore, Cinzia Costa, Paolo Prontera, Francesca Bisulli, Lorenzo Muccioli, Giuseppe Damante

**Affiliations:** 1 Department of Medicine, University of Udine, Piazzale Kolbe 4, 33100 Udine, Italy; lorenzo.allegri@uniud.it (L.A.); federica.baldan@asfo.sanita.fvg.it (F.B.); catia.mio@uniud.it (C.M.); 2 Laboratory of Experimental Neurology, Department of Medicine and Surgery, University of Perugia, 06123 Perugia, Italy; valentina.imperatore@unipg.it; 3 Section of Neurology and Laboratory of Experimental Neurology, Department of Medicine and Surgery, University of Perugia, 06123 Perugia, Italy; cinzia.costa@unipg.it; 4 Medical Genetics Unit, S. Maria della Misericordia Hospital, 06123 Perugia, Italy; pprontera@hotmail.com; 5 IRCCS Istituto delle Scienze Neurologiche di Bologna, Reference Network for Rare and Complex Epilepsies (EpiCARE), 40126 Bologna, Italy; francesca.bisulli@unibo.it (F.B.); lorenzo.muccioli@gmail.com (L.M.); 6 Department of Biomedical and Neuromotor Sciences, University of Bologna, 40126 Bologna, Italy; 7 Institute of Medical Genetics, Academic Hospital “Azienda Sanitaria Universitaria Friuli Centrale”, 33100 Udine, Italy

**Keywords:** Lafora, SINEUP, amylase

## Abstract

**Background/Objectives:** Lafora disease is a fatal and progressive neurodegenerative disorder characterized by the accumulation of insoluble polyglucosan inclusions, known as Lafora bodies, due to impaired glycogen metabolism. Therapeutic strategies aimed at reducing intracellular glycogen accumulation represent a promising approach to mitigating disease progression. This study aimed to evaluate the feasibility of promoting Lafora body degradation by increasing the protein levels of human pancreatic amylase, a glycogen-degrading enzyme, through the SINEUP approach. **Methods:** Two SINEUP constructs specifically targeting human pancreatic amylase were designed and tested in continuous tumor-derived cell lines of central nervous system origin, as well as in primary fibroblasts obtained from a patient with Lafora disease. Human pancreatic amylase protein and mRNA levels were assessed to determine the specificity of SINEUP-mediated regulation. Enzymatic activity assays were performed to evaluate functional protein upregulation, and intracellular glycogen content was measured in patient-derived fibroblasts. **Results:** Both SINEUP constructs significantly increased human pancreatic amylase protein expression without affecting mRNA levels, confirming a post-transcriptional mechanism of action. The elevated protein levels were associated with a significant increase in enzymatic activity. In primary fibroblasts derived from a Lafora disease patient, enhanced amylase expression correlated with a marked reduction in intracellular glycogen content. **Conclusions:** These findings provide proof of concept that SINEUP-mediated upregulation of glycogen-degrading enzymes may represent a viable therapeutic strategy to counteract Lafora body accumulation. Further studies are warranted to assess the efficacy, safety, and translational potential of this approach, particularly in relevant animal models of Lafora disease.

## 1. Introduction

Lafora disease (LD) is an ultra-rare, severe, autosomal recessive neurodegenerative disorder belonging to the group of progressive myoclonus epilepsies [1]. It affects previously healthy individuals, with disease onset typically occurring in late childhood or early adolescence (mean age ~13 years) [2]. The disease course is characterized by drug-resistant epilepsy accompanied by myoclonus, ataxia, and cognitive deterioration, ultimately leading to loss of autonomy and premature death at a median of 6 and 11 years after disease onset, respectively [2]. At present, treatment is purely symptomatic, and no disease-modifying therapies are available.

LD is caused by homozygous or compound heterozygous pathogenic mutations in one of two genes, *EPM2A* or *EPM2B* (i.e., *NHLRC1*), which contribute independently and equally to the pathogenesis of the disease. Most pathogenic variants result in loss of protein function, and include splice-site, missense, nonsense, insertion, and small intragenic deletion mutations [1,3]. LD occurs worldwide but shows a higher incidence in Mediterranean countries (including Italy, France, and Spain), North Africa, and Central/South Asia (Pakistan and India), with an estimated prevalence of fewer than one case per million individuals [4]. Pathologically, LD is characterized by the accumulation of abnormal glycogen aggregates consisting of excessively long, poorly branched, and hyperphosphorylated chains. These aggregates precipitate into insoluble polyglycosan inclusions (Lafora bodies, LBs), which accumulate in multiple tissues, including the liver, skeletal muscle, and central nervous system (CNS) [5]. In the brain, LB accumulation triggers neuroinflammation and promotes neurodegeneration [6]. The two LD-associated genes encode laforin (*EPM2A*) [7] and malin (*NHLRC1*) [8], which function together as a glycogen quality-control system. Laforin is a glucan phosphatase, while malin is an E3 ubiquitin ligase. When glycogen becomes excessively long and poorly branched, laforin binds the abnormal chains and recruits malin, which limits further synthesis and promotes chain degradation. Loss of this checkpoint results in accumulation of insoluble, hyperphosphorylated glycogen aggregates that precipitate as Lafora bodies (LBs) despite normal glycogen synthase and branching enzyme activity [9]. Given the central role of LBs in determining the LD phenotype, therapeutic strategies aimed at reducing or eliminating these aggregates have gained increased attention [10]. In recent years, studies have explored the use of antibody-enzyme fusion proteins capable of penetrating the cell membrane and delivering human pancreatic α-amylase to degrade LBs in vivo [11]. These findings support pancreatic α-amylase-based approach as a promising strategy to mitigate disease pathology in LD, which currently remains an untreatable and fatal disease. 

SINEUPs represent a class of recently discovered antisense long non-coding RNAs known for their ability to increase the translation of target mRNAs [12]. Their molecular structure consists of an antisense domain called the binding domain (BD) that recognizes the specific sequence of the target mRNA, and a domain folded into a tertiary structure responsible for enhancing translation, called the effector domain (ED) [13]. The use of SINEUP has been proven to rescue haploinsufficient gene dosage in a medakafish model of a human disorder, leading to amelioration of the disease phenotype [14]. Bon and colleagues also demonstrated how the SINEUP design specific to the target of interest is able to increase FXN protein expression levels in a cellular model of Friedreich’s ataxia [15]. These studies demonstrate that the use of SINEUP is effective in inducing endogenous overexpression of genes of interest, partially bypassing the issues associated with mechanisms based on exogenous overexpression [16].

Given these premises, the aim of this study is to investigate the potential use of SINEUPs as an innovative therapeutic strategy to upregulate the human pancreatic α-amylase and trigger the degradation of LBs in vitro.

## 2. Materials and Methods

### 2.1. Cell Lines

In this study, we used a line of fibroblasts from a patient with LD harboring mutation c.386C>A; p.Pro129His (GM08935) and three CNS-derived tumor cell lines (SH-SY5Y, U87 and CCF-STTG1). GM08935 and U87 cells were grown in DMEM (Euroclone S.p.A, Milano, Italy) supplemented with 20% FBS (GM08935) or 10% (U87) FBS (Gibco; Thermo Fisher Scientific, Inc., Waltham, MA, USA) and 100 I.U./mL penicillin and 100 μg/mL streptomycin (Merck Millipore, Burlington, MA, USA). SH-SY5Y cells were cultured in DMEM-F12 medium (Euroclone S.p.A, Milano, Italy) while CCF-STTG1 cells were cultured in RPMI-1640 (Euroclone S.p.A, Milano, Italy), both supplemented with 10% FBS (Gibco; Thermo Fisher Scientific, Inc., Waltham, MA, USA), and 100 I.U./mL penicillin and 100 μg/mL streptomycin (Merck Millipore, Burlington, MA, USA). Finally, we also used a murine astrocyte culture (C8D1A; CRL-2541, ATCC) that was grown in DMEM (Gibco; Thermo Fisher Scientific, Inc., Waltham, MA, USA) supplemented with 10% FBS (Gibco; Thermo Fisher Scientific, Inc., Waltham, MA, USA), and 100 I.U./mL penicillin and 100 μg/mL streptomycin (Gibco; Thermo Fisher Scientific, Inc., Waltham, MA, USA). Cells were cultured in a humidified incubator (5% CO_2_ and 95% air at 37 °C) (Eppendorf AG, Hamburg, Germany).

### 2.2. RNA Extraction, Quantification and Gene Expression Assay

Total RNA from GM08935, SH-SY5Y, U87, and CCF-STTG1 cells and from C8D1A murine cells was extracted using the RNeasy Mini Kit according to the manufacturer’s instructions (Qiagen, Hilden, Germany). RNA was quantified using the Qubit RNA HS assay (Thermo Fisher Scientific, Waltham, MA, USA) in a Qubit 4.0 Fluorometer. A total of 1 μg RNA from all cell lines was reverse transcribed to cDNA as already described [17]. Quantitative PCR was performed using PowerUP Sybr green master mix (Thermo Fisher Scientific, Waltham, MA, USA) on the QuantStudio3 system (Applied Biosystems, Waltham, MA, USA). The QuantStudio Design and Analysis software v1.5.0 (Applied Biosystems) was used to calculate mRNA levels with the 2^−ΔΔCt^ method, and ß-actin was used as a reference. All experiments were performed in triplicate.

### 2.3. SINEUPs Design and Cloning

SINEUPs for the human and murine amylase gene were generated as already described [15] using pcDNA 3.1(−)-Δ5′-AS Uchl1 as a backbone, removing the region specific for Uchl1 (BD) and retaining the effector domain. The specific binding domain (BD) for amylase mRNA was designed in antisense orientation to the sequence common to the main isoforms of human amylase. Two BDs were designed, a long one (−40/+4 bp) called SINEUP 1 and a short one (−14/+4 bp) called SINEUP 2, both overlapping the AUG of the gene of interest. Oligonucleotides were annealed and cloned into the recipient plasmid.

### 2.4. SINEUPs Transfection and Amylase Overexpression

Cells were plated in 6-well plates the day before transfection at 60% confluency (4 × 10^5^ cells/well) and transfected with 1 μg SINEUPs encoding plasmids using Lipofectamine LTX (ThermoFisher Scientific, Waltham, MA, USA) following the manufacturer’s instructions. Then, 1 µg of pcDNA 3.1 (ThermoFisher Scientific, Waltham, MA, USA) empty plasmid was transfected as a negative control. To over-express α-amylase protein as a positive control, TrueClone *AMY2A* cDNA cloned in pCMV6-AC, which contains a full open reading frame of the human *AMY2A* gene (Origene, Rockville, MD, USA), was transfected. Cells were collected 48 h after treatments.

### 2.5. Protein Extraction and Western Blot

Total protein extraction and Western blotting were performed as described previously [18,19]. In brief, proteins were electrophoresed on SDS-PAGE and then transferred to nitrocellulose membranes (GE Healthcare, Little Chalfont, UK), saturated with 5% non-fat dry milk in PBS/0.1% Tween 20. The membranes were then incubated overnight with mouse monoclonal anti-amylase antibody or mouse anti-vinculin antibody (Santa Cruz Biotechnology, Dallas, TX, USA). The day after, membranes were incubated with anti-mouse immunoglobulin coupled to peroxidase (Merck KGaA) for 2 h. Blots were developed using UVITEC Alliance LD (UVITec Limited, Cambridge, UK) with the SuperSignal Technology (Thermo Scientific Inc., Waltham, MA, USA).

### 2.6. Amylase Enzymatic Activity and Quantification of Intracellular Glycogen Content

The evaluation of intracellular amylase enzyme activity was performed using an amylase activity assay colorimetric kit (Merck Millipore, Burlington, MA, USA) following the manufacturer’s protocol. In brief, 10^6^ cells per experimental condition were mechanically lysed by pipetting in 500 μL of kit buffer. The cell lysate was centrifuged at 13,000× *g* for 10 min to remove insoluble material. Then, 50 µL of sample was placed in each well of a 96-well plate, and 100 µL of master reaction mix was added to each well. After 2–3 min of incubation at 25 °C, the initial absorbance was read at 405 nm. Subsequent absorbance measurements were taken until the next measurement was lower than the previous one. The highest measurement in the series was considered for each sample. In addition, the method described here [20] was used to quantify intracellular glycogen content. 5 × 10^6^ cells for each experimental condition were mechanically lysed, the glycogen contained in the lysates was precipitated by adding 50 μL of 0.4 M sodium sulfate and then adding 950 μL of 100% ethanol and centrifuging at 16,000× *g* for 25 min at 4 °C. Once the pellets were re-suspended in water, the glycogen was quantified using a glycogen assay kit (Merck Millipore, Burlington, MA, USA), measuring the color intensity at 570 nm.

### 2.7. Animals

The Epm2aR240X mouse model of Lafora disease was generated as previously described [21,22]. Animals were bred at Centro di Ricerca Preclinica at the University of Perugia, where they were maintained in individually housed cages under a 12:12 light/dark cycle at a constant temperature of 23 °C, with food and water available ad libitum. All animal experiments complied with European Directive 2010/63/EU and were conducted according to protocols approved by the Animal Care and Use Committee of the University of Perugia (authorization n°08/2018-UT).

### 2.8. Collection of Brain Regions

Mice were sacrificed by cervical dislocation. The brain was collected and immersed in ice-cold artificial cerebrospinal fluid (ACSF) containing (in mM): 126 NaCl, 2.5 KCl, 1.2 MgCl_2_, 1.2 NaH_2_PO_4_, 2.4 CaCl_2_, 10 glucose, and 25 NaHCO_3_, bubbled with 95% O_2_ and 5% CO_2_, pH 7.4. Brain regions, including the hippocampus, striatum, cortex, and cerebellum, were dissected from the whole brain. All procedures were carried out on ice, and the isolated regions were immediately transferred into tubes containing D-PBS (Gibco; Thermo Fisher Scientific, Inc., Waltham, MA, USA) supplemented with protease inhibitors (Merck Sigma-Aldrich; Darmstadt, Germany). To obtain a positive control for the α-amylase detection assays—given the pancreatic origin of the enzyme—the pancreas was also collected and processed under the same conditions. All tissues were stored at −80 °C. We collected samples from five knock-in (KI) laforin mice carrying the R240X mutation in the *EPM2A* gene.

### 2.9. Protein Extraction from Murine Tissues

Protein lysates were prepared from murine tissues using a Total Lysis Buffer (TLB) composed of 50 mM Tris-HCl pH 8, 120 mM NaCl, 5 mM EDTA, 1% Triton X-100, and 1% NP-40. Immediately before use, the buffer was supplemented with protease inhibitors (1:100), 1 mM NaF, 1 mM NaOrthovanadate, 0.5 mM PMSF, and 1 mM DTT.

Tissues were briefly rinsed in ice-cold PBS containing protease inhibitors (1:100). Each sample was trimmed into ~30–40 mg pieces and mechanically minced on ice. Approximately 100 µL of freshly supplemented TLB was added, and the homogenate was transferred to microcentrifuge tubes. Samples were further disrupted using a 1 mL syringe, avoiding bubble formation, and vortexed five times. Lysates were incubated for 30 min at 4 °C and centrifuged at 14,000× *g* for 10 min at 4 °C. When necessary, supernatants were passed through a 0.45 µm syringe filter to remove residual connective tissue. The clarified supernatant was collected and stored at −20 °C, while pellets were retained at −20 °C for potential downstream analyses.

### 2.10. Statistical Analysis

All data were expressed as means ± SD, and significances were analyzed with either Student’s *t*-test or one-way ANOVA, both performed using GraphPad Prism 10.0.0 (GraphPad Software, Inc., San Diego, CA, USA). The *n* value indicates the number of biological replicates for each experiment.

## 3. Results

### 3.1. Basal Expression of Pancreatic α-Amylase in Continuous Cell Lines

**Figure 1:**
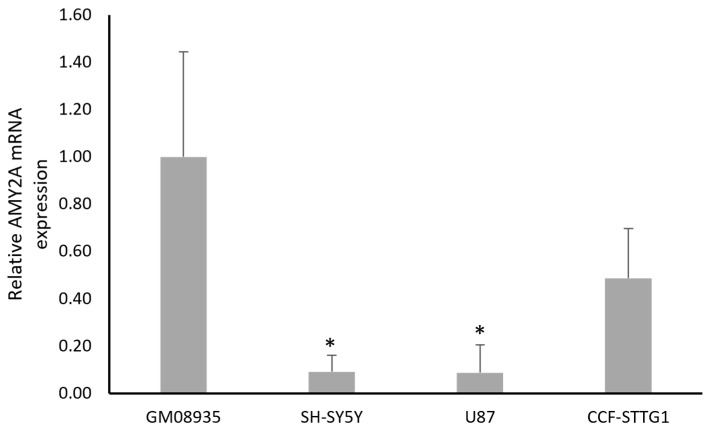
mRNA levels for *AMY2A* in fibroblasts from a patient with LD (GM08935) or in cancer cells derived from the CNS (SH-SY5Y, U87 and CCF-STTG1). Values represent relative expression quantified using the 2^−ΔΔCt^ method, normalized to β-actin, with GM08935 cells set to 1 and mRNA levels in cancer cells were expressed as fold change. *n* = 3. * *p* < 0.05.

As the first step in our experimental approach, we evaluated the expression levels of the pancreatic α-amylase gene (*AMY2A*) in different continuous cell lines. Three lines of tumors derived from CNS (SH-SY5Y, U87 and CCF-STTG1) and a line of fibroblasts obtained from a patient with LD carrying a pathogenic mutation in the *NHLRC1* gene (GM08935) were used as experimental models. The mRNA was extracted and expression levels were measured by real-time PCR after reverse transcription. Figure 1 shows that GM08935 cells derived from patients express higher levels of *AMY2A*, almost 10 times higher than SH-SY5Y and U87. CCF-STTG1 cells also appear to express reduced levels of amylase, although with a smaller difference (about half) that is not statistically significant.

**Figure 2:**
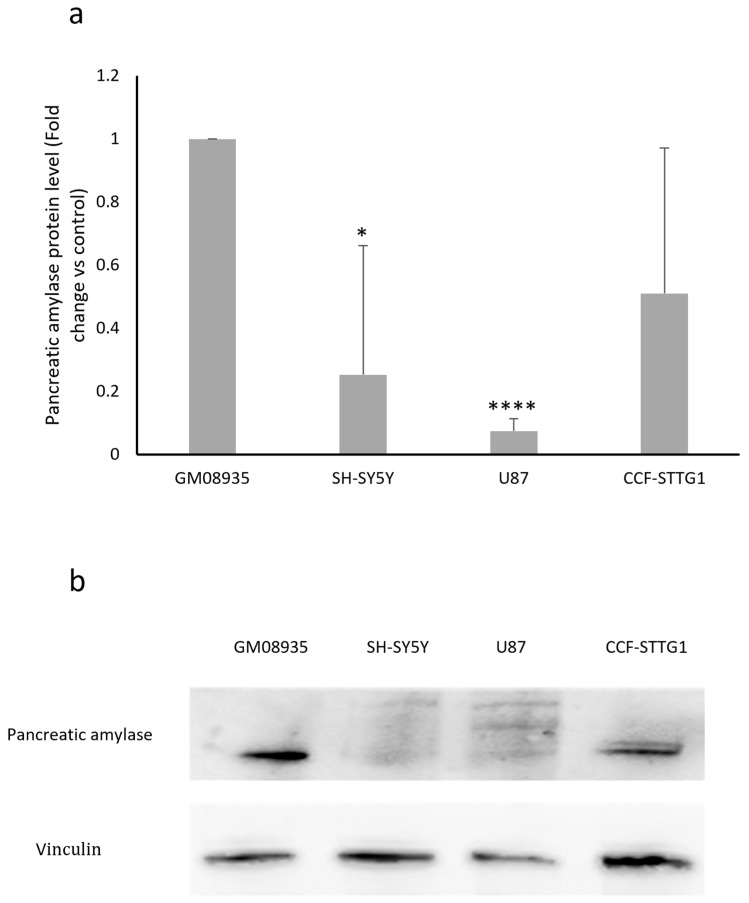
(**a**), Protein levels for pancreatic α-amylase in fibroblasts from a patient with LD (GM08935) or in cancer cells derived from the CNS (SH-SY5Y, U87 and CCF-STTG1). Pancreatic amylase levels were normalized to vinculin levels in each sample. GM08935 amylase protein level was set to 1 and protein levels in cancer cells were expressed as fold change. *n* = 3. * *p* < 0.05, **** *p* < 0.0001. (**b**), Representative image of a Western blot testing human pancreatic amylase levels in GM08935, SH-SY5Y, U87, and CCF-STTG1 cells.

In order to confirm the results obtained regarding *AMY2A* gene expression, pancreatic α-amylase protein levels were evaluated using Western blot analysis. As shown in the graph in Figure 2a, the basal expression levels of amylase in the different cell lines essentially reflect what was observed in terms of mRNA expression. GM08935 is confirmed to be the cell line with the highest expression of α-amylase when compared with the tumor-derived lines.

### 3.2. Effects of AMY2A-Specific SINEUPs in CNS-Derived Tumor Cells and LD Fibroblasts

**Figure 3:**
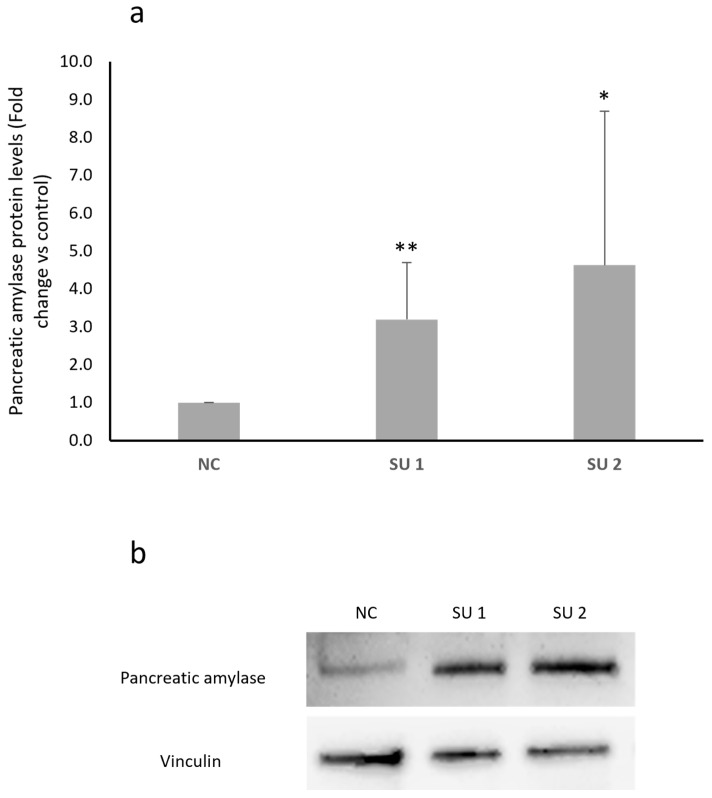

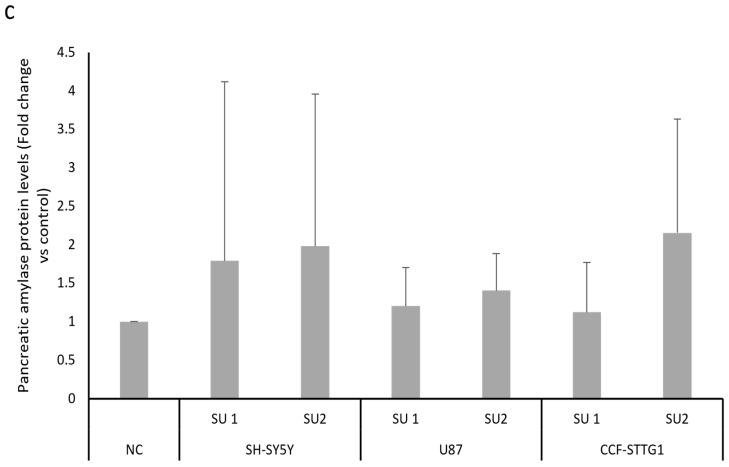
(**a**) Western blot densitometric analysis in LD GM08935 cells. Cells were treated for 48 h with 1 μg of two different vectors, each carrying a different SINEUP (SU 1 or SU 2), or with the empty vector as a negative control (NC). Pancreatic amylase levels were normalized to vinculin levels in each sample. Negative control (NC) amylase protein level was set to 1 and protein levels in treated cells were expressed as fold change. *n* = 6. * *p* < 0.05, ** *p* < 0.01. (**b**) Representative image of a Western blot testing human pancreatic amylase levels after SINEUP transfection in GM08935. (**c**) Western blot analysis of pancreatic amylase protein levels in CNS-derived cancer cell lines. Cells were treated for 48 h with 1 μg of two different vectors, each carrying a different SINEUP (SU 1 or SU 2), or with the empty vector as a negative control (NC). Pancreatic amylase levels were normalized to vinculin levels in each sample. Amylase protein level of NC was set to 1 and protein levels in treated cells were expressed as fold change.

After designing two SINEUPs specific for human amylase mRNA, cells were transfected with different concentrations based on data already available in the literature [16]. Cells were treated with 1 μg of plasmids each containing a SINEUP or with an empty plasmid as a negative control (NC), and the proteins were extracted after 48 h of treatment. The results obtained from the Western blot analysis of pancreatic amylase expression differed depending on the cell line. LD cells showed a statistically significant increase in amylase protein levels after administration of both SINEUPs, as shown in Figure 3a. SINEUP 1 was able to determine a 1.5-fold increase, while SINEUP 2 determined an even greater increase of approximately 4-fold in pancreatic amylase levels in this cell line. When treated with the two SINEUPs, SH-SY5Y, U87, and CCF-STTG1 cells gave similar results. Panel b of Figure 3 shows an increase in amylase protein levels in all three lines, with SINEUP 2 once again appearing to cause a greater increase. However, unlike what was observed in LD cells, these cell lines showed extreme variability in response between experiments, which resulted in a lack of statistical significance.

### 3.3. Amylase Enzymatic Activity After SINEUPs Transfection

**Figure 4:**
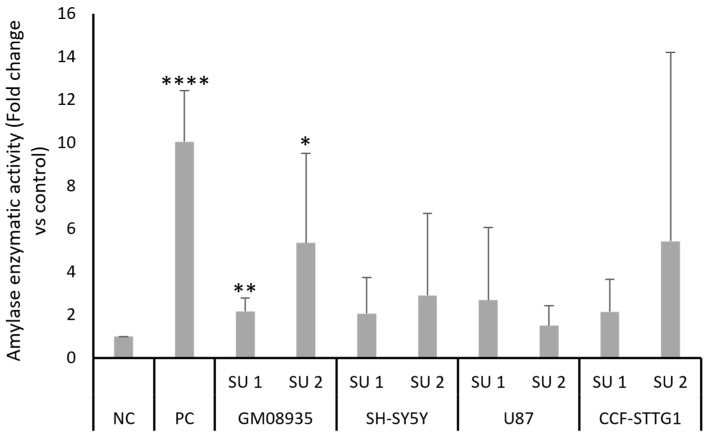
Colorimetric evaluation of amylase enzyme activity. GM08935, SH-SY5Y, U87, and CCF-STTG1 cells were treated for 48 h with 1 µg of empty plasmid (NC) or plasmid containing a SINEUP (SU 1 or SU 2). As a positive control, GM 08935 cells were transfected with a plasmid containing human pancreatic α-amylase (PC). Results are expressed as arbitrary units, with the negative control (NC) set at 1. *n* = 6. * *p* < 0.05, ** *p* < 0.01, **** *p* < 0.0001.

In order to assess whether the increased level of pancreatic amylase protein expression following treatment with SINEUPs also translated into an actual increase in its enzymatic activity in the treated cell lines, the ability of amylase to cleave a substrate was tested. Cells were transfected with 1 μg of two plasmids containing the two SINEUPs or NC. Furthermore, a positive control (PC), such as an expression vector containing the complete *AMY2A* coding region, was used. Moreover, 48 h after transfection, the cells were collected and lysed, and endogenous amylase activity was tested using a colorimetric assay. Figure 4 shows the results of the colorimetric test for amylase activity; in agreement with the observations made in the Western blots, treatment with SINEUPs of LD GM 08935 cells induced an increase in amylase enzyme activity. This increase ranges from approximately two to six times for SINEUP 1 and SINEUP 2, respectively. An increase in the ability of amylase to cleave the substrate was also observed in the other cell lines, but once again, marked variability resulted in a high standard deviation, which did not lead to a statistically significant variation.

### 3.4. Quantification of Intracellular Glycogen Content

**Figure 5:**
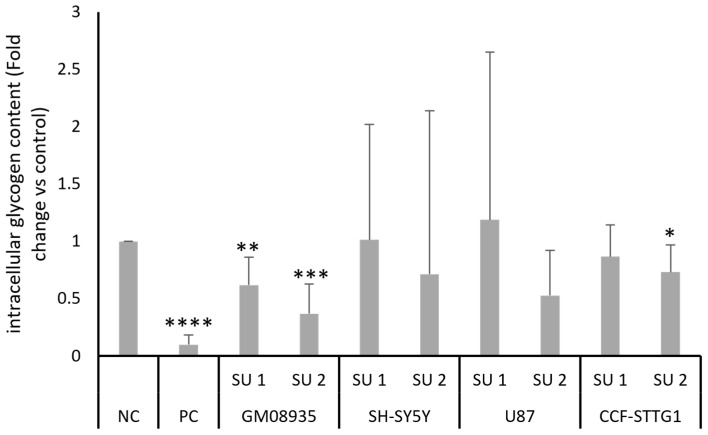
Colorimetric evaluation of intracellular glycogen content. GM08935, SH-SY5Y, U87, CCF-STTG1 cells were treated for 48 h with 1 µg of NC, PC or plasmid containing a SINEUP (SU 1 or SU 2). Results are expressed as arbitrary units, with NC set at 1. *n* = 5. * *p* < 0.05, ** *p* < 0.01, *** *p* < 0.001, **** *p* < 0.0001.

After demonstrating the increase in pancreatic amylase protein levels and the corresponding increase in its enzymatic activity, the amount of glycogen contained in cell lines following treatment with SINEUPs was tested. Cells were transfected for 48 h with two SINEUPs, PC or NC. After two days of treatment, intracellular glycogen content was quantified using a colorimetric assay. Results shown in Figure 5 show a statistically significant decrease in glycogen levels in GM08935 cells treated with the two SINEUPs specific for pancreatic α-amylase. Similar to what was observed for amylase enzyme activity, although there was an underlying trend of reduced glycogen levels in the other cell lines as well, the high variability meant that this reduction was not statistically significant, with the exception, this time, of treatment with SINEUP 2 in CCF-STTG1 cells.

### 3.5. Pancreatic Alfa-Amylase Expression in Murine Tissues

Western blot analysis revealed robust α-amylase expression in the pancreas of 3-month-old KI-laforin mice, consistent with its known pancreatic origin. Lower but clearly detectable levels of the enzyme were also observed across all examined brain regions, including cortex, cerebellum, striatum, and hippocampus. Densitometric quantification normalized to the pancreatic signal confirmed the presence of α-amylase throughout the CNS, indicating that KI-laforin mice express measurable amounts of the enzyme in neural tissues. All analyses were performed on three independent biological replicates (Supplementary Figure S1).

## 4. Discussion

Lafora disease (LD) is a rapidly progressive neurodegenerative epilepsy for which no disease-modifying therapies are currently available. Increasing evidence indicates that Lafora bodies (LBs), insoluble polyglucosan inclusions, play a central pathogenic role by directly contributing to neuronal dysfunction and neurodegeneration [23,24]. Consequently, therapeutic strategies aimed at reducing LB burden have emerged as a rational and promising approach. Among LB-targeting strategies, promoting the degradation of already accumulated inclusions may offer a distinct advantage, as diagnosis typically occurs after substantial pathological damage has developed. Pancreatic α-amylase has been shown to hydrolyze the abnormal glycogen that constitutes LBs, and enzyme-delivery approaches have demonstrated efficacy in reducing LB load and improving disease-associated phenotypes in LD models [25,26]. On this basis, we investigated whether SINEUP technology could be exploited to enhance endogenous α-amylase expression as an alternative means to promote LB clearance. SINEUPs act at the translational level, increasing protein output without altering mRNA abundance and thereby allowing controlled upregulation of endogenous targets [12,16]. In the present study, two SINEUP constructs targeting human pancreatic α-amylase were tested in different cellular models. In fibroblasts derived from an LD patient, both constructs significantly increased α-amylase protein levels, resulting in enhanced enzymatic activity and a measurable reduction in intracellular glycogen content, although, with regard to this last result, it is not possible to establish whether the decrease in glycogen observed is due to the degradation of LB or whether it is attributable to the degradation of other intracellular polyglucosans. These findings demonstrate that SINEUP-mediated upregulation of α-amylase is functionally effective in a disease-relevant in vitro model. By contrast, CNS-derived tumor cell lines showed a less consistent response, with increased protein levels displaying high inter-experimental variability and failing to reach statistical significance. This difference is likely attributable to the markedly lower basal expression of α-amylase in these cells. Because SINEUPs enhance translation rather than transcription [14], a minimal level of target mRNA appears to be required for detectable activity, limiting their effectiveness in systems with very low endogenous expression. To further explore this aspect, we designed two SINEUP constructs targeting the murine amylase transcript and tested them in the C8D1A mouse astrocyte cell line. Neither construct yielded a measurable increase in amylase protein abundance or enzymatic activity. These negative findings are consistent with the very low endogenous amylase expression in cultured murine astrocytes and support the notion that SINEUP activity requires a minimum basal level of the target transcript. In contrast, Western blotting of laforin knock-in (KI) mice detected amylase in whole-brain lysates and in discrete regions, cortex, cerebellum, striatum, and hippocampus, albeit at lower levels than in the pancreas (Supplementary Figure S1). This pattern, which accords with prior in vivo reports of pancreatic α-amylase in astrocytes and neurons [27,28]. These findings provide biological context regarding the endogenous presence of α-amylase in the murine CNS and indicate that neural tissues express measurable amounts of the enzyme, although at substantially lower levels than the pancreas. While these observations do not constitute functional evidence, they outline the baseline expression landscape in vivo and may help inform future studies aimed at evaluating SINEUP-mediated modulation of α-amylase in animal models. Although the magnitude of α-amylase upregulation achieved in this study was limited, the results provide proof of concept for the feasibility of this approach. Given the previously demonstrated in vivo efficacy of SINEUP-based strategies in neurodegenerative disease models, further studies are warranted to assess whether sustained enhancement of α-amylase expression can promote meaningful LB clearance in LD. In conclusion, this work supports SINEUP-mediated upregulation of pancreatic α-amylase as a potential therapeutic strategy for LD. With further optimization and validation in relevant in vivo models, this approach may contribute to the development of novel interventions targeting the pathological accumulation of glycogen in Lafora disease. 

## Data Availability

The original contributions presented in this study are included in the article/Supplementary Materials. Further inquiries can be directed to the corresponding author.

## Supplementary Materials

The following supporting information can be downloaded at: https://www.mdpi.com/article/10.3390/genes17030321/s1, Figure S1: Expression of pancreatic alpha-amylase in the brain of KI-laforin mice. Western blot analyses were performed on lysates from pancreas and specific brain regions (cortex, cerebellum, striatum, hippocampus) of 3-month-old KI-laforin mice. Amylase protein was readily detected in pancreas and was also present at lower levels in all examined brain regions. Densitometric quantification, normalized to the positive control (pancreas), confirms detectable amylase expression across the CNS, supporting the suitability of this model for in vivo testing of murine amylase-specific SINEUPs.

## References

[1] Nitschke F., Ahonen S.J., Nitschke S., Mitra S., Minassian B.A. (2018). Lafora Disease—From Pathogenesis to Treatment Strategies. Nat. Rev. Neurol..

[2] Pondrelli F., Muccioli L., Licchetta L., Mostacci B., Zenesini C., Tinuper P., Vignatelli L., Bisulli F. (2021). Natural History of Lafora Disease: A Prognostic Systematic Review and Individual Participant Data Meta-Analysis. Orphanet J. Rare Dis..

[3] Mata-Garrido J., Tapia O., Casafont I., Berciano M.T., Cuadrado A., Lafarga M. (2018). Persistent Accumulation of Unrepaired DNA Damage in Rat Cortical Neurons: Nuclear Organization and ChIP-Seq Analysis of Damaged DNA. Acta Neuropathol. Commun..

[4] Orphanet: Lafora Disease. https://www.orpha.net/en/disease/detail/501.

[5] Gentry M.S., Guinovart J.J., Minassian B.A., Roach P.J., Serratosa J.M. (2018). Lafora Disease Offers a Unique Window into Neuronal Glycogen Metabolism. J. Biol. Chem..

[6] Mitra S., Gumusgoz E., Minassian B.A. (2022). Lafora Disease: Current Biology and Therapeutic Approaches. Rev. Neurol..

[7] Chan E.M., Ackerley C.A., Lohi H., Ianzano L., Cortez M.A., Shannon P., Scherer S.W., Minassian B.A. (2004). Laforin Preferentially Binds the Neurotoxic Starch-like Polyglucosans, Which Form in Its Absence in Progressive Myoclonus Epilepsy. Hum. Mol. Genet..

[8] Chan E.M., Young E.J., Ianzano L., Munteanu I., Zhao X., Christopoulos C.C., Avanzini G., Elia M., Ackerley C.A., Jovic N.J. (2003). Mutations in *NHLRC1* Cause Progressive Myoclonus Epilepsy. Nat. Genet..

[9] Hejazi M., Fettke J., Kötting O., Zeeman S.C., Steup M. (2010). The Laforin-like Dual-Specificity Phosphatase SEX4 from Arabidopsis Hydrolyzes Both C6- and C3-Phosphate Esters Introduced by Starch-Related Dikinases and Thereby Affects Phase Transition of Alpha-Glucans. Plant Physiol..

[10] Gentry M.S., Markussen K.H., Sun R.C., Vander Kooi C.W., Noebels J.L., Avoli M., Rogawski M.A., Vezzani A., Delgado-Escueta A.V. (2024). Treating Lafora Disease with an Antibody-Enzyme Fusion. Jasper’s Basic Mechanisms of the Epilepsies.

[11] Austin G.L., Simmons Z.R., Klier J.E., Rondon A., Hodges B.L., Shaffer R., Aziz N.M., McKnight T.R., Pauly J.R., Armstrong D.D. (2019). Central Nervous System Delivery and Biodistribution Analysis of an Antibody-Enzyme Fusion for the Treatment of Lafora Disease. Mol. Pharm..

[12] Carrieri C., Cimatti L., Biagioli M., Beugnet A., Zucchelli S., Fedele S., Pesce E., Ferrer I., Collavin L., Santoro C. (2012). Long Non-Coding Antisense RNA Controls Uchl1 Translation through an Embedded SINEB2 Repeat. Nature.

[13] Zucchelli S., Fasolo F., Russo R., Cimatti L., Patrucco L., Takahashi H., Jones M.H., Santoro C., Sblattero D., Cotella D. (2015). SINEUPs Are Modular Antisense Long Non-Coding RNAs That Increase Synthesis of Target Proteins in Cells. Front. Cell. Neurosci..

[14] Indrieri A., Grimaldi C., Zucchelli S., Tammaro R., Gustincich S., Franco B. (2016). Synthetic Long Non-Coding RNAs [SINEUPs] Rescue Defective Gene Expression In Vivo. Sci. Rep..

[15] Bon C., Luffarelli R., Russo R., Fortuni S., Pierattini B., Santulli C., Fimiani C., Persichetti F., Cotella D., Mallamaci A. (2019). SINEUP Non-Coding RNAs Rescue Defective Frataxin Expression and Activity in a Cellular Model of Friedreich’s Ataxia. Nucleic Acids Res..

[16] Espinoza S., Scarpato M., Damiani D., Managò F., Mereu M., Contestabile A., Peruzzo O., Carninci P., Santoro C., Papaleo F. (2020). SINEUP Non-Coding RNA Targeting GDNF Rescues Motor Deficits and Neurodegeneration in a Mouse Model of Parkinson’s Disease. Mol. Ther..

[17] Molteni E., Baldan F., Damante G., Allegri L. (2023). GSK2801 Reverses Paclitaxel Resistance in Anaplastic Thyroid Cancer Cell Lines through MYCN Downregulation. Int. J. Mol. Sci..

[18] Molteni E., Baldan F., Damante G., Allegri L. (2024). Dihydrotanshinone I Exhibits Antitumor Effects via β-Catenin Downregulation in Papillary Thyroid Cancer Cell Lines. Sci. Rep..

[19] Allegri L., Baldan F., Roy S., Aubé J., Russo D., Filetti S., Damante G. (2019). The HuR CMLD-2 Inhibitor Exhibits Antitumor Effects via MAD2 Downregulation in Thyroid Cancer Cells. Sci. Rep..

[20] Nitschke S., Petković S., Ahonen S., Minassian B.A., Nitschke F. (2020). Sensitive Quantification of α-Glucans in Mouse Tissues, Cell Cultures, and Human Cerebrospinal Fluid. J. Biol. Chem..

[21] Ganesh S., Delgado-Escueta A.V., Sakamoto T., Avila M.R., Machado-Salas J., Hoshii Y., Akagi T., Gomi H., Suzuki T., Amano K. (2002). Targeted Disruption of the *Epm2a* Gene Causes Formation of Lafora Inclusion Bodies, Neurodegeneration, Ataxia, Myoclonus Epilepsy and Impaired Behavioral Response in Mice. Hum. Mol. Genet..

[22] Burgos D.F., Sciaccaluga M., Worby C.A., Zafra-Puerta L., Iglesias-Cabeza N., Sánchez-Martín G., Prontera P., Costa C., Serratosa J.M., Sánchez M.P. (2023). *Epm2a*^R240X^ Knock-in Mice Present Earlier Cognitive Decline and More Epileptic Activity than *Epm2a*^−/−^ Mice. Neurobiol. Dis..

[23] Duran J., Brewer M.K., Hervera A., Gruart A., Del Rio J.A., Delgado-García J.M., Guinovart J.J. (2020). Lack of Astrocytic Glycogen Alters Synaptic Plasticity but Not Seizure Susceptibility. Mol. Neurobiol..

[24] Sinha P., Verma B., Ganesh S. (2021). Trehalose Ameliorates Seizure Susceptibility in Lafora Disease Mouse Models by Suppressing Neuroinflammation and Endoplasmic Reticulum Stress. Mol. Neurobiol..

[25] Nikaido T., Austin J., Stukenbrok H. (1971). Studies in Myoclonus Epilepsy. 3. The Effects of Amylolytic Enzymes on the Ultrastructure of Lafora Bodies. J. Histochem. Cytochem..

[26] Brewer M.K., Uittenbogaard A., Austin G.L., Segvich D.M., DePaoli-Roach A., Roach P.J., McCarthy J.J., Simmons Z.R., Brandon J.A., Zhou Z. (2019). Targeting Pathogenic Lafora Bodies in Lafora Disease Using an Antibody-Enzyme Fusion. Cell Metab..

[27] Byman E., Schultz N., Blom A.M., Wennström M., Netherlands Brain Bank (2019). A Potential Role for α-Amylase in Amyloid-β-Induced Astrocytic Glycogenolysis and Activation. J. Alzheimers Dis..

[28] Byman E., Schultz N., Fex M., Wennström M., Netherlands Brain Bank (2018). Brain Alpha-Amylase: A Novel Energy Regulator Important in Alzheimer Disease?. Brain Pathol..

