# Supplementary material for: SINEUP-Mediated Overexpression of Endogenous α-Amylase as a Therapeutic Approach in Lafora Disease
Source: Genes (Basel). 2026 Mar 16;17(3):321. doi: 10.3390/genes17030321 (PMC13026089; doi:10.3390/genes17030321)
Supplement: Supplementary file 1 [file genes-17-00321-s001.zip › genes-4168415-supplementary.pdf]

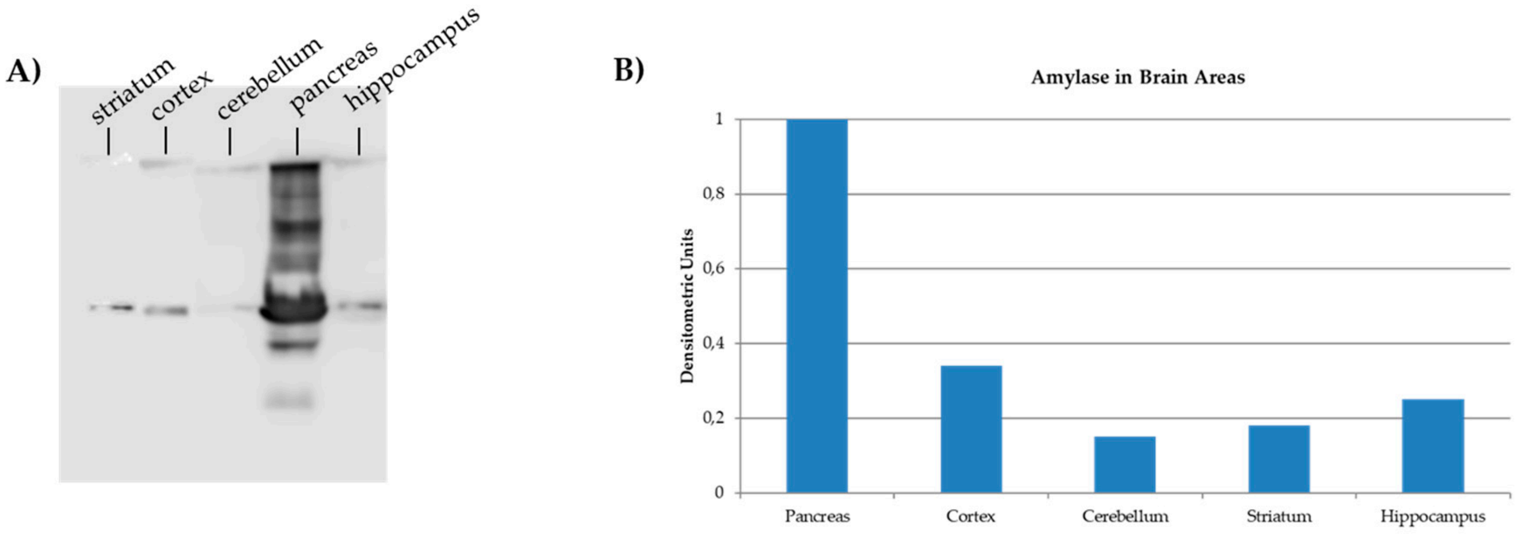

Suppl. Fig.S1. Expression of pancreatic alpha-amylase in the brain of KI-laforin mice. Western blot analyses were performed on lysates from pancreas and specific brain regions (cortex, cerebellum, striatum, hippocampus) of 3-month-old KI-laforin mice. Amylase protein was readily detected in pancreas and was also present at lower levels in all examined brain regions. Densitometric quantification, normalized to the positive control (pancreas), confirms detectable amylase expression across the CNS, supporting the suitability of this model for in vivo testing of murine amylase-specific SINEUPs.
